# Forest fragmentation and edge effects impact body condition, fur condition and ectoparasite prevalence in a nocturnal lemur community

**DOI:** 10.1093/conphys/coae042

**Published:** 2024-07-02

**Authors:** Daniel Hending, Heriniaina Randrianarison, Niaina Nirina Mahefa Andriamavosoloarisoa, Christina Ranohatra-Hending, Grainne McCabe, Sam Cotton, Marc Holderied

**Affiliations:** Department of Biology, University of Oxford, 11A Mansfield Road, Oxford OX1 3SZ, UK; School of Biological Sciences, University of Bristol, 24 Tyndall Avenue, Bristol BS8 1TH, UK; Institute of Conservation Science & Learning, Bristol Zoological Society, Clifton, Bristol BS8 3HA, UK; Zoologie et Biodiversité Animale, Université d’Antananarivo, BP 906 Antananarivo 101, Madagascar; Zoologie et Biodiversité Animale, Université d’Antananarivo, BP 906 Antananarivo 101, Madagascar; School of Biological Sciences, University of Bristol, 24 Tyndall Avenue, Bristol BS8 1TH, UK; Institute of Conservation Science & Learning, Bristol Zoological Society, Clifton, Bristol BS8 3HA, UK; Institute of Conservation Science & Learning, Bristol Zoological Society, Clifton, Bristol BS8 3HA, UK; Wilder Institute, Calgary Zoo, 1300 Zoo Road NE, Calgary, AB T2E 7V6, Canada; Institute of Conservation Science & Learning, Bristol Zoological Society, Clifton, Bristol BS8 3HA, UK; School of Biological Sciences, University of Bristol, 24 Tyndall Avenue, Bristol BS8 1TH, UK

**Keywords:** Cheirogaleidae, fur condition scores, habitat degradation, lemur conservation, Lepilemuridae, live trapping, physiological health, Sahamalaza

## Abstract

Forest fragmentation and edge effects are two major threats to primate populations. Primates inhabiting fragmented landscapes must survive in a more degraded environment, often with lower food availability compared to continuous forests. Such conditions can have deleterious effects on animal physiological health, yet some primates thrive in these habitats. Here, we assessed how forest fragmentation and associated edge effects impact three different components of physiological health in a nocturnal primate community in the Sahamalaza-Iles Radama National Park, northwest Madagascar. Over two periods, 6 March 2019–30 October 2019 and 10 January 2022–17 May 2022, we collected data on body condition, fur condition scores and ectoparasite prevalence for 125 *Mirza zaza*, 51 *Lepilemur sahamalaza*, 27 *Cheirogaleus medius* and 22 *Microcebus sambiranensis* individuals, and we compared these metrics between core and edge areas of continuous forest and fragmented forest. Body condition scores for all species varied between areas, with a positive response to fragmentation and edge effects observed for *M. zaza* and *L. sahamalaza* and a negative response for *C. medius* and *M. sambiranensis*. Fur condition scores and ectoparasite prevalence were less variable, although *M. zaza* and *L. sahamalaza* had a significantly negative response to fragmentation and edge effects for these two variables. Interestingly, the impacts of fragmentation and edge effects on physiological health were variable-specific. Our results suggest that lemur physiological responses to fragmentation and edge effects are species-specific, and body condition, fur condition and ectoparasite prevalence are impacted in different ways between species. As other ecological factors, including food availability and inter/intraspecific competition, likely also influence physiological health, additional work is required to determine why certain aspects of lemur physiology are affected by environmental stressors while others remain unaffected. Although many nocturnal lemurs demonstrate resilience to fragmented and degraded habitats, urgent conservation action is needed to safeguard the survival of their forest habitats.

## Teaser

We assessed how forest fragmentation and edge effects impact three physiological health components in a nocturnal primate community in northwest Madagascar. Physiological responses to fragmentation and edge effects were both species- and variable-specific. Our results highlight the resilience of some primate species to environmental change.

## Abbreviations

HW, head width
GLMM, generalized linear mixed model
SMI, scaled mass index

## Introduction

Deforestation and habitat fragmentation are major causes of animal population decline and species extirpation (Sodhi *et al.,* 2004; Haddad *et al.,* 2015). The ongoing destruction of tropical forests is a particular conservation concern, as more than 50% of the world’s terrestrial animal species depend on tropical forest habitat for their survival (Wilson, 1988; Roberts *et al.,* 2018). Further, more than half of the world’s remaining tropical forest habitat is now severely fragmented and degraded (Chazdon *et al.,* 2009; Taubert *et al.,* 2018), resulting in lower resource availability (Laurance and Bierregaard, 1997), enhanced predation and hunting pressures (Irwin *et al.,* 2009; Harrison *et al.,* 2016) and increased parasite exposure for resident animal populations (Lafferty and Kuris, 2005) in comparison with intact primary forest. As animal physiology is strongly regulated by environmental factors (Rangel-Negrín *et al.,* 2009), deforestation and fragmentation can have profoundly negative impacts on the physical health and well-being of wild animal populations (Walsh *et al.,* 1993). Although the sensitivity of animals to environmental change is species-specific (Keinath *et al.,* 2017), habitat fragmentation and degradation have been observed to have deleterious effects on the body condition (Cardozo and Chiaraviglio, 2008), parasite load (Schwitzer *et al.,* 2011) and stress level (Martínez-Mota *et al.,* 2007) of many animal populations. Consequently, the identification and mitigation of these physiological effects are now principal components of animal conservation (Wikelski and Cooke, 2006; Acevedo-Whitehouse and Duffus, 2009; Ellis *et al.,* 2012).

The physiological responses of animals to habitat degradation and fragmentation can reveal important information concerning their vulnerability, resilience and flexibility to environmental change (Romero, 2004; Kobbe *et al.,* 2011), and they are therefore useful tools for detecting and monitoring potential conservation concerns (Cooke *et al.,* 2013; Rakotoniaina *et al.,* 2016). The effect of habitat-related factors on animal physiology is arguably most studied in primates, as their slow life histories and strict dependence on tropical forests make them particularly sensitive to environmental change (Isabirye-Basuta and Lwanga, 2008; Balestri *et al.,* 2014). Such environmental changes include forest fragmentation and anthropogenic disturbance, which have been linked to declines in body condition in some primates, such as ring-tailed lemurs, olive baboons and Milne-Edwards’s sifakas (Eley *et al.,* 1989; Sauther *et al.,* 2006; Dunham *et al.,* 2008; Singleton *et al.,* 2015). Similarly, habitat degradation has been associated with increased parasite prevalence in colobus monkeys, redtail guenons and mangabeys (Gillespie *et al.,* 2005; Chapman *et al.,* 2006; Gillespie and Chapman, 2008; Mbora and McPeek, 2009). Fur condition, which provides an indication of nutritional deficiencies, endocrine functions and disease, has also been observed to vary between primate populations in pristine and degraded habitats (Berg *et al.,* 2009; Jolly, 2009a; Crawford *et al.,* 2015). Many studies that have investigated the relationship between physiology and habitat have focused on only one single aspect of physiology (Rakotoniaina *et al.,* 2016). As several explanatory factors are often attributable to a process as complex as physiological change, a single variable is often not enough to predict how a species responds to its surroundings (e.g. Johns and Skorupa, 1987; Milton, 1996; Sauther *et al.,* 2002; Junge *et al.,* 2011), despite the potential use of these indicators as a tool for species conservation. A multifaceted approach is therefore needed to ultimately determine the physiological response of an animal to forest fragmentation and habitat degradation (Rakotoniaina *et al.,* 2016).

Madagascar is well known for its high species biodiversity, much of which is endemic (Goodman and Benstead, 2005). However, Madagascar is also noted for its high rates of deforestation (Harper *et al.,* 2007; Goodman *et al.,* 2018; Vieilledent *et al.,* 2018), which has become a serious threat for lemurs, Madagascar’s flagship group of animals (Schwitzer *et al.,* 2013; Goodman, 2022). The fragmentation of Madagascar’s forests is a particularly severe threat for lemur populations, as the key functional traits of many species, including small body size, make it challenging for them to cross large open spaces between isolated fragments (Wilmé *et al.,* 2006; Olivieri *et al.,* 2008), and over 95% of lemur species are now listed as threatened on the International Union for Conservation of Nature Red List (IUCN, 2020). Due to their imperiled status, some studies have already investigated the physiological responses of lemurs to forest fragmentation and degradation, and as with many other primates (Martínez-Mota *et al.,* 2007; Rimbach *et al.,* 2014; Chaves *et al.,* 2019), some lemurs appear to be affected by these changes to their habitats. For example, deterioration of body and fur conditions has been observed in ring-tailed lemurs in response to habitat degradation (Sauther *et al.,* 2006; Jolly, 2009b), while the health status of the Indri is reportedly lower in highly disturbed forest areas (Junge *et al.,* 2011). Forest fragmentation and degradation have also been recognized to cause health decline in diademed sifakas (Irwin *et al.,* 2010) and increased parasite prevalence in red-collared brown lemurs, grey mouse lemurs and blue-eyed black lemurs (Raharivololona and Ganzhorn, 2009; Schwitzer *et al.,* 2010; Lazdane *et al.,* 2014). In contrast, ectoparasite prevalence in mouse lemurs has been observed to decrease in fragmented and edge habitats (Kiene *et al.,* 2020). However, forest fragmentation and degradation have also been observed to have little effect on the health of some other lemur species (e.g. *Hapalemur griseus*: Grassi, 2001; *Propithecus verreauxi*: Loudon and Sauther, 2013; *Microcebus murinus* and *Cheirogaleus medius*: Rakotoniaina *et al.,* 2016), suggesting that lemur responses to changes in habitat are species-specific. The effects of forest fragmentation, habitat degradation and anthropogenic disturbance on lemur health remain little studied, and this is something that needs to be addressed to conserve Madagascar’s remaining lemur populations (Schwitzer *et al.,* 2013).

Here, we investigated the impacts of forest fragmentation and forest edge effects on the body condition, fur condition and ectoparasite prevalence in a nocturnal lemur community in the Sahamalaza-Iles Radama National Park (hereafter referred to as SIRNP), northwest Madagascar, using a multi-method approach. We specifically chose to focus on nocturnal species for this study as there is evidence of resilience and adaptability to habitat change among nocturnal lemurs (Lehman *et al.,* 2016; Knoop *et al.,* 2018; Hending, 2021), and our study site harbors four sympatric nocturnal species, all of which are threatened with extinction (Randriatahina *et al.,* 2020; Reuter and Schwitzer, 2020; Blanco *et al.,* 2020a; Blanco *et al.,* 2020b). Our study species were the Vulnerable northern giant mouse lemur (*Mirza zaza*), the Critically Endangered Sahamalaza sportive lemur (*Lepilemur sahamalaza*), the Endangered Sambirano mouse lemur (*M. sambiranensis*) and the Vulnerable fat-tailed dwarf lemur (*C. medius*). The specific aims of our study were to:

1) Investigate how the body condition of each species varies between continuous and fragmented forest and between forest edge and core areas.
2) Investigate how the fur condition scores of each species vary between continuous and fragmented forest and between forest edge and core areas.
3) Investigate how ectoparasite prevalence within each species varies between continuous and fragmented forest and between forest edge and core areas.

Based on the different ecologies, dietary niches and tolerance of habitat degradation of our study species (Hending *et al.,* 2024; Hending *et al.,* in press) and the findings of previous investigations (Singleton *et al.,* 2015; Rakotoniaina *et al.,* 2016; Springer and Kappeler, 2016), we hypothesized that the physiological responses of the four lemur species to forest fragmentation would be species-specific, and we predicted that body condition, fur condition scores and ectoparasite prevalence would vary between continuous and fragmented forest habitats and between forest edge and core areas. The physiological differences between continuous and fragmented forest and between edge and core habitats were expected to be most profound in *L. sahamalaza* due to the more specialized ecology and narrower feeding niches of sportive lemurs compared to the Cheirogaleidae (Ganzhorn, 1993). We also predicted significant differences for *C. medius*, as their activity is highly dependent on immediate environmental conditions as a result of their extended torpor schedules (Dausmann *et al.,* 2004; Fietz and Dausmann, 2006). In contrast, we anticipated that these differences would be much less profound in *M. zaza* and *M. sambiranensis* due to their generalist ecology and tolerance of degraded habitat types typical of the Cheirogaleidae (Lehman *et al.,* 2016).

## Materials and Methods

### Ethical declarations

All research complied with UK Home Office policies when working with animals, and all research adhered to the legal requirements of Madagascar. Research in the SIRNP was permitted by Madagascar National Parks (Permit numbers 245/19 and 124/22—MEEF/SG/DGGE/DAPRNE/SCBE.Re). We consulted the Code of Best Practices for Field Primatology when planning all methods undertaken in this study.

### Study site

**Figure 1:**
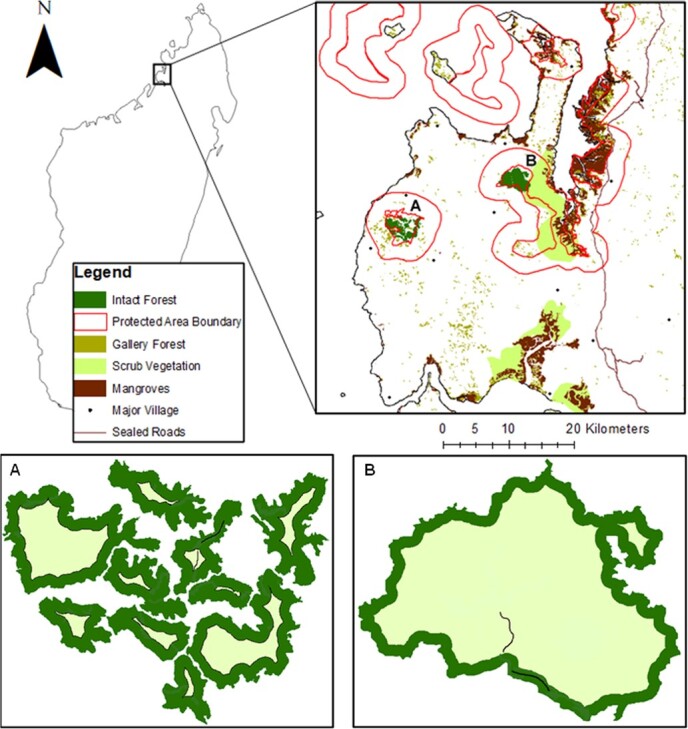
The SIRNP and the location of Ankarafa forest (**A**) and Anabohazo forest (**B**). The red Protected Area Boundary lines represent the protected area boundary itself (inner) and a 3-km buffer zone (outer). Dark green areas represent edge forest (≤165 m from the edge), light green areas represent core forest ((>165 m from the edge) and black lines represent trapping transects in (A) and (B). Figure created in ArcMap, with a scale of 1:7 000 000 for Madagascar and a scale of 1:350 000 for the zoomed SIRNP panel.

SIRNP is a 26 000 Ha National Park located in Madagascar’s Sofia Region (latitude: 14°04′S—14°37′S, longitude: 47°52′E—48°04′E) (Fig. 1). In addition to National Park status, SIRNP has been designated as a UNESCO Biosphere Reserve since 2007 (Rode *et al.*, 2013), yet despite this protected status it has undergone heavy deforestation and habitat fragmentation in recent years (Seiler *et al.*, 2010; Hending *et al.*, 2017a). Although some isolated forests and tracts of scrub and gallery forest remain within SIRNP, large areas of the protected area are now characterized by anthropogenic savannah and grasslands (Penny *et al.*, 2014). SIRNP is located in the Sambirano domain, an area of North West Madagascar characterized by transitional sub-humid forests comprised of both evergreen and deciduous flora, of which many species are locally endemic (Koechlin, 1972; Du Puy and Moat, 1998). SIRNP’s climate is hot, sub-humid and highly seasonal, with a humid wet season (November–April) and a cooler dry season (May–October) (Hending *et al.*, 2017b). The mean temperature range of SIRNP is 20.6–32.0°C, with an extreme temperature range of 13.2–39.1°C and a mean annual precipitation of ~ 1600 mm (Schwitzer *et al.*, 2007).

SIRNP is an ideal location to investigate the effect of forest fragmentation and edge-effects on the physiological health of nocturnal lemurs. This is firstly because two forests remain within SIRNP: the 1169 Ha Anabohazo Forest, a continuous forest that has undergone relatively little anthropogenic disturbance (Randriatahina *et al.*, 2014), and the fragmented Ankarafa forest, a 1020 Ha forest comprising many fragments of varying size, shape and forest edge-core ratios (Supplementary File S1). Secondly, vegetation plots measuring tree species diversity, size and forest structural diversity (Hending *et al.*, 2023a), and transects measuring the variation of microclimatic and abiotic variables (temperature, humidity and light intensity) from the forest edge into the forest core (Mandl *et al.*, 2023) have already been conducted to establish the edge boundary of the forests of SIRNP. This edge boundary has been determined to be 165 m (Mandl *et al.*, 2023). Thirdly, Anabohazo and Ankarafa are separated by only ~ 25 km of open grassland, providing an opportunity to study physiological responses of lemurs to forest fragmentation on a localized scale, where environmental and habitat covariates can be easily controlled for.

### Trapping

We conducted all fieldwork between 6 March 2019 and 15 May 2022, a period that included both the wet (November–April) and dry (May–October) seasons in SIRNP. We used a live-trapping technique to capture *M. zaza, M. sambiranensis* and *C. medius* individuals for physiological assessment. Prior to trapping, we established two ~ 600 m line transects within each forest (four transects total) upon which we set our live-traps. In each forest, one transect went exclusively through the edge area of the forest (≤ 165 m from forest edge) and the other exclusively through the forest core area (> 165 m from the edge), to ensure that we captured lemurs in both edge and core habitat. We surveyed the two forests separately; trapping in Anabohazo took place 6 March 2019–25 July 2019 and 10 January 2022–04 March 2022 whilst trapping in Ankarafa was carried out 5 August 2019–30 October 2019 and 10 March 2022–15 May 2022. No sampling took place in 2020 and 2021 due to the COVID-19 pandemic and the closure of Madagascar’s border. The Ankarafa trapping lines were situated within the 70 Ha Ankarafa I fragment. We used a total of 56 live-traps (40 Sherman XLF15 and 16 Tomahawk Size 12), which were divided equally between the edge and core trapping lines (*N* = 28 traps per transect). The traps were positioned at ~ 20 m intervals along the transect lines and were fixed at varying heights in the tree (1.5–4.5 m), where they were fastened to a branch with twine. During survey periods, we conducted trapping 3 days per week on consecutive nights (Monday–Wednesday), where traps were set at ~17:00 and baited with fresh banana. We then left the traps for the duration of the night and returned the next morning at ~07:00 to check for captures. The total number of trap nights were Anabohazo Core *N* = 53, Anabohazo Edge *N* = 53, Ankarafa Core *N* = 38, Ankarafa Edge *N* = 38. As it is difficult to capture sportive lemurs in live traps (Ravaoarimanana *et al.*, 2004), we captured *L. sahamalaza* individuals by hand from their day sleeping sites (as in Schmid and Ganzhorn, 1996; Zinner *et al.*, 2003). Using protective animal-handling gauntlets, we also captured a number of *L. sahamalaza* individuals by hand whilst out on nocturnal walks in the forest.

### Body measurements

We handled all captured lemurs with protective gloves at the site of the trap during trap checks, or for *L. sahamalaza*, at the exact location where they were captured immediately after capture. One team member trained in the handling of primates conducted all handling and measurements. Once the lemurs were restrained, we identified their sex and we measured their weight using a mammal-holding bag and spring scales (Pesola 500 g and 2.5 kg, Schindellegi, Switzerland). We then took body measurements of the lemurs using high-precision measuring calipers (Sealey Vernier, Bury, UK) (following Rasoloarison *et al.*, 2000; Rakotoniaina *et al.*, 2016). These measurements were as follows:

- Head length: the distance from the nose tip to the distalmost point of the head.
- Body length: the distance from the base of the head to the distalmost point of the body (tail base).
- Tail length: the distance from the tail base to the distalmost vertebra.
- Total length: the sum of the head length, body length and tail length.
- Tail base circumference: the circumference of the tail base at its widest point.
- Head width: the distance between the two most-lateral points of the zygomatic arches (bizygomatic breadth).

We recorded the capture location of all lemurs using a handheld GPS (eTrex 30, Garmin, Olathe, Kansas, USA), and we used an electronic shaver (P2, iClipper, Ningbo Zhejiang, China) to mark the tails of all captured lemurs with an individual-specific stripe pattern. The tail patterns coupled with our body measurement data and capture location enabled us to successfully identify all lemur individuals if they were recaptured.

We later used a scaled mass index (SMI) to estimate the individual body condition of our captured lemurs, which uses a distinct body size measurement and the individual’s body mass to reflect internal energy reserves and therefore overall body condition (Peig and Green, 2009). We chose to use head width (HW) to calculate our SMI values as this measurement has the highest correlation with body mass for small mammalian species and reflects internal energy reserves (Peig and Green, 2009), and this method has also successfully been applied to cheirogaleid lemurs (Rakotoniaina *et al.*, 2016). We used the following formula to calculate the SMI for all of our captured individuals (*i*):

SMI*_i_* = M*_i_* (HW_0_/HW*_i_*) ^bSMA^.

HW_0_ is the mean HW for each of our study species (*M. zaza* = 34.23 mm, *L. sahamalaza* = 40.02 mm, *C. medius* = 32.44 mm, *M. sambiranensis* = 21.77 mm) and the species-specific scaling component bSMA is the slope of the standardized major axis regression of body mass to HW. We obtained the bSMA values (*M. zaza* = 2.377, *L. sahamalaza* = 0.872, *C. medius* = 7.100, *M. sambiranensis* = 1.837) using the RMA software (Bohonak and Van der Linde, 2004), with M*_i_* assigned as the Y-variable and HW*_i_* as the X-variable. Following Peig and Green (2009), all M*_i_*, HW*_0_* and HW*_i_* values were logged to calculate bSMA values.

### Fur quality assessments

In addition to body condition, we assessed the fur condition scores of our captured lemurs. Using the lemur fur score system developed by Berg *et al.* (2009), we visually inspected the body fur and tail fur of the lemurs. We assigned a health-score to both the body fur and tail fur based on fur thickness and glossiness using the score criteria developed by Berg *et al.* (2009) and the photos of each health score category that they included in their study (summarized in Table 1). These fur quality measurements are an additional measurement of physiological health and a proxy of well-being and have since been applied to other studies (Jolly, 2009a). The same team of researchers took all body measurements, graded the fur of all individuals and collected the data of fur quality (two researchers and one local guide) to avoid inter-observer bias between different team members (Schüßler *et al.*, 2024).

**Table 1:** The body and tail fur scores (BS and TS) and the criteria used to assign them, adapted from the scoring system of Berg *et al.* (2009), for our captured lemur individuals in the SIRNP, 6 March 2019–15 May 2022

**Fur score**	**Criteria**
BS0—Good	Complete, fluffy fur cover. Fur is glossy (one or two small holes may be present)
BS1—Rough	Complete fur cover but slightly thinned or shaggy (with one or two holes possibly present)
BS2—Holes	Good or rough fur but with bald patches or holes covering up to 25% of the body
BS3—Ragged	Base of hairs visible down to the skin on up to 50% of the body. 25–50% of fur has holes
BS4—Sheared	Fur less than half of normal thickness on over half of the body
BS5—Bald	More than 50% of the fur is missing (bald patches)
Tail fur score	Criteria
TS0—Good	Fully furred and bushy tail over its entire length
TS1—Pointy	Tails may taper towards the end (less bushy at the tip). No bare parts but thinned over less than half of the length
TS2—Thin	Up to 50% of the tail thinned to less than half of the normal fur thickness
TS3—Ragged	Partly hairless, but less than half of the tail is bare
TS4—Sheared	Tail fur less than half of normal thickness on over half of the entire length
TS5—Bald	Fur is missing completely on over 50% of the tail’s total length

### Ectoparasite prevalence

We assessed all captured lemurs for the presence of ectoparasites, as ectoparasite presence can be a good indicator of poor health in primates (Sauther *et al.*, 2002). After we recorded body measurements and fur scores of our captured lemurs, we visually inspected exposed areas of skin (inside of ears, genital area etc.) on the lemur’s bodies for ticks. We then used a lice comb to rake through the full length of fur on the lemur’s dorsal region, whilst also checking for lice and fleas in the fur of the abdomen and limbs (as in Kiene *et al.*, 2020) with a magnifying glass. To ensure standardization across individuals, we used only two full strokes of the lice-comb from head to the tail-tip to extract lice and fleas from the fur. We identified the type of ectoparasite to three broad groups using a reference from the literature (Zohdy and Durden, 2016): (i) ticks (Ixodida) and mites (Acari), (ii) lice (Phthiraptera) and (iii) fleas (Siphonaptera). We noted whether each type of ectoparasite was present or absent within each sampled individual and we noted the number of each ectoparasite that we could find (as in Takahata *et al.*, 1998; Sauther *et al.*, 2002). We were unable to collect specimens of the ectoparasites for genus/species identification as microscopes were not available at the study site, and specimens decompose quickly and would therefore be unusable by the time they reached a laboratory facility (alcohol and formalin for preservation were also not available at the study site). We released all captured lemur individuals back into the capture-tree after we had finished our ectoparasite assessments; the full process to measure all investigated variables took ~ 5 minutes per individual.

### Data analysis

We performed all statistical analyses in R (R Core Team, 2022), using an alpha value of 0.05. Prior to analysis, we averaged any data for individuals that were captured multiple times (recaptured), so that we had only one data entry per individual. To assess the impacts of forest fragmentation and edge effects on body condition, we used linear mixed models (LMMs, one for each species) where the fixed factor was forest area (Anabohazo core, Anabohazo edge, Ankarafa core and Ankarafa edge), with season (dry 2019, dry 2022, wet 2019 or wet 2022) and sex as random factors. Although we always ran both edge and core trapping lines simultaneously at the same time, we included season as a random factor due to variations in food availability within SIRNP’s forests (Mandl *et al.*, 2018; Hending *et al.*, 2023b) and the seasonal behavior typical of cheirogaleids and lepilemurids (Fietz and Ganzhorn, 1999; Dausmann *et al.*, 2004; Mandl *et al.,* 2018), both of which may influence body condition throughout the year. We included sex as a random factor due to potential sex-specific differences in activity (Rasoazanabary, 2006; Seiler *et al.*, 2019), torpor bouts (Schmid and Kappeler, 1998; Schmid, 1999), reproductive states (Radespiel *et al.*, 2022) and stress levels (Rakotoniaina *et al.*, 2016; Bethge *et al.*, 2022), all of which can profoundly influence mass. For *M. sambiranensis*, body-condition was only compared between Anabohazo core and Anabohazo edge as this species was not known to occur within Ankarafa until after this study had commenced (Hending *et al.*, 2022a) and we did not capture any individuals of this species in Ankarafa. SMI values were log-transformed for LMMs to improve model fit (Rakotoniaina *et al.*, 2016). LMMs were performed using the R package ‘lme4’ (Bates *et al.*, 2015). As the fur condition scores datasets were ordinal categorical values, we compared how body and tail fur varied between the four forest areas using Kruskal–Wallis tests and Dunn’s *post hoc* tests with Bonferroni correction. We performed these tests using the R package ‘FSA’ (Ogle *et al.,* 2023). For ectoparasite presence analyses, we used the unaveraged version of the data as parasite prevalence varied between individual recapture events. Ectoparasite data was zero-inflated and of non-normal distribution, so we log(*x* + 1) transformed all values. To compare the counts of each of the three parasite groups between sites for each species, we performed generalized linear mixed models (GLMMs) with Poisson distributions on the transformed data. This taxon-based approach was based on similar studies of ectoparasite prevalence in nocturnal lemurs (Kiene *et al.*, 2020; Durden *et al.*, 2021; Marquès Gomila *et al.*, 2023). As for the prior models, we included season (for both sampling years) and sex as random factors, but we also included individual ID and body mass as random factors to account for pseudoreplication and potential correlation between lemur size and parasite prevalence (Durden *et al.*, 2021). To compare the number of instances of multiple ectoparasite group infection between sites, we used GLMMs with a binomial family and a logit link function, and the random factors season, sex, individual ID and body mass.

**Table 2:** The mean (±SD) body mass (g), HW (mm) and total length (mm) of four species of nocturnal lemurs in the core and edge areas of the Anabohazo and Ankarafa forests, Sahamalaza-Iles Radama National Park, north west Madagascar

		Site			
Species	Measurement	Anabohazo core	Anabohazo edge	Ankarafa core	Ankarafa edge
*Mirza zaza*	*N*	42	27	35	21
	Mass (g)	198.04 ± 45.81	195.96 ± 48.08	245.8 ± 33.72	233.05 ± 48.33
	HW (mm)	36.93 ± 3.68	33.81 ± 3.22	32.41 ± 1.70	32.38 ± 2.42
	Total length (mm)	501.90 ± 35.41	507.20 ± 30.45	496.14 ± 27.24	506.86 ± 29.29
*Lepilemur sahamalaza*	*N*	12	8	15	16
	Mass (g)	677.25 ± 60.94	678.50 ± 89.61	712.93 ± 94.14	680.00 ± 85.67
	HW (mm)	46.67 ± 9.75	40.63 ± 2.34	37.73 ± 3.49	36.88 ± 3.81
	Total length (mm)	541.42 ± 25.07	525.75 ± 21.52	512.60 ± 21.74	499.38 ± 30.54
*Cheirogaleus medius*	*N*	4	5	9	9
	Mass (g)	294.00 ± 10.37	267.60 ± 19.89	232.89 ± 48.38	187.22 ± 52.98
	HW (mm)	32.25 ± 1.09	31.80 ± 1.47	32.89 ± 0.87	32.44 ± 1.57
	Total length (mm)	465.25 ± 6.38	456.60 ± 9.60	459.11 ± 12.24	456.78 ± 18.16
*Microcebus sambiranensis*	*N*	7	15	0	0
	Mass (g)	37.86 ± 4.94	36.60 ± 7.70	-	-
	HW (mm)	20.57 ± 1.29	22.33 ± 2.30	-	-
	Total length (mm)	249.86 ± 13.64	250.73 ± 27.00	-	-

Data were collected between 6 March 2019 and15 May 2022.

## Results

### Body condition

We captured a total of 225 lemur individuals during our study, not including recaptures. We caught a total of 125 *M. zaza*, 51 *L. sahamalaza*, 27 *C. medius* and 22 *M. sambiranensis*. The morphology of each species varied among the two surveyed forests and among the core and edge areas of each forest (Table 2). Mean mass for *M. zaza* was 216.84 g (116–312 g), 688.80 g (426–886 g) for *L. sahamalaza*, 236.85 g (116–317 g) for *C. medius* and 37.00 g (25–52 g) for *M. sambiranensis*, whilst mean total length was 502.26 mm for *M. zaza*, 517.29 mm for *L. sahamalaza*, 458.78 mm for *C. medius* and 250.45 mm for *M. sambiranensis*. Body condition for *M. zaza* (estimate = 2.274, *df* = 1.341, *t* = 33.910, *P* = 0.006), *L. sahamalaza* (estimate = 2.783, *df* = 1.499, *t* = 74.430, *P* = 0.001) and *C. medius* (estimate = 2.479, *df* = 3.451, *t* = 29.509, *P* < 0.001) varied significantly between the four forest areas sampled. *M. zaza* body condition was significantly higher in Ankarafa core and edge areas in comparison to both Anabohazo core (*P* < 0.001) and edge (*P* < 0.001) areas and higher in Anabohazo edge in comparison to core (*P* = 0.031) (Supplementary File S2). *L. sahamalaza* body condition was significantly lower in Anabohazo core in comparison to Ankarafa core (*P* = 0.008) and Ankarafa edge (*P* = 0.021), whilst there was no difference between the core and edge areas of Anabohazo (*P* = 1.000). For *C. medius*, body condition only varied significantly between Anabohazo edge (highest scaled body mass index) and Ankarafa edge (lowest scaled body mass index) (*P* = 0.031). *M. sambiranensis* body condition was significantly higher in the core area of Anabohazo in comparison to the edge area (estimate = 1.619, *df* = 1.847, *t* = 49.171, *P* < 0.001) (Supplementary File S2). The effects of sex on body condition appear to be significant in *M. zaza* (SD = 0.028), *C. medius* (SD = 0.033) and *M. sambiranensis* (SD = 0.020) but not for *L. sahamalaza* (SD = 0.000) (Supplementary File S2).

### Fur condition scores

Fur condition scores for *M. zaza* did not vary significantly between any of the sites (*H* = 6.168, *df* = 3, *P* = 0.104, Table 3). However, *M. zaza* tail fur condition scores did vary significantly between sites (*H* = 29.696, *df* = 3, *P* < 0.001), with Anabohazo core tail fur condition scores significantly lower than in the other three areas (Table 3). Fur condition scores for *L. sahamalaza* varied significantly between sites (*H* = 13.617, *df* = 3, *P* = 0.003), with Ankarafa edge fur scores being significantly higher than in the other three areas (Table 3). Tail fur condition scores for *L. sahamalaza* also varied significantly between sites (*H* = 10.756, *df* = 3, *P* = 0.013), with Ankarafa edge tail fur scores being significantly higher than in the core areas of both Anabohazo and Ankarafa (Table 3). Fur condition scores for *C. medius* did not vary significantly between any of the sites (*H* = 6.050, *df* = 3, *P* = 0.109). However, *C. medius* tail fur condition scores did vary significantly between sites (*H* = 9.620, *df* = 3, *P* = 0.022), with Anabohazo core and edge tail fur condition scores being significantly lower than in Ankarafa edge (Table 3). Both fur (*W* = 37.000, *df* = 1, *P* = 0.239) and tail fur (*W* = 40.500, *df* = 1, *P* = 0.369) condition did not vary significantly between Anabohazo core and edge for *M. sambiranensis* (Table 3).

**Table 3:** The mean (±SD) fur and tail fur scores of four species of nocturnal lemurs in the core and edge areas of the Anabohazo and Ankarafa forests, Sahamalaza-Iles Radama National Park, north west Madagascar

		Site			
Species	Measurement	Anabohazo Core	Anabohazo Edge	Ankarafa Core	Ankarafa Edge
*Mirza zaza*	*N*	42	27	35	21
	Fur score	0.55 ± 0.66^a^	0.96 ± 0.84^a^	0.60 ± 0.49^a^	0.90 ± 0.87^a^
	Tail score	0.62 ± 0.65^a^	1.70 ± 1.18^b^	1.60 ± 0.87^b^	1.76 ± 1.15^b^
*Lepilemur sahamalaza*	*N*	12	8	15	16
	Fur score	0.08 ± 0.28^a^	0.00 ± 0.00^a^	0.20 ± 0.40^a^	0.88 ± 0.93^b^
	Tail score	0.50 ± 0.65^a^	0.63 ± 0.48^ab^	0.60 ± 0.61^a^	1.75 ± 1.25^b^
*Cheirogaleus medius*	*N*	4	5	9	9
	Fur score	0.25 ± 0.43^a^	0.40 ± 0.49^a^	0.78 ± 0.63^a^	1.22 ± 0.79^a^
	Tail score	0.00 ± 0.00^a^	0.00 ± 0.00^a^	0.67 ± 0.67^ab^	1.44 ± 1.17^b^
*Microcebus sambiranensis*	*N*	7	15	0	0
	Fur score	0.43 ± 0.49^a^	0.80 ± 0.65^a^	-	-
	Tail score	0.43 ± 0.49^a^	0.73 ± 0.68^a^	-	-

Values with the same superscript letter within a row are not significantly different from each other in following Kruskal–Wallis tests and Dunn’s post-hoc tests (corrected for multiple comparisons, following Bonferroni correction). Data were collected between 6 March 2019 and 15 May 2022.

### Ectoparasite prevalence

In *M. zaza*, tick/mite (Estimate = 0.013, *df* = 1.515, *t* = 0.517, *P* = 0.671), lice (Estimate = −0.008, *df* = 0.065, *t* = −0.124, *P* = 0.971) and flea (estimate = 0.029, *df* = 25.193, *t* = 1.613, *P* = 0.119) prevalence did not vary significantly across study sites (Supplementary File S3). However, there were some significant differences in pairwise comparisons (Table 4); *M. zaza* in core areas of both forests had significantly lower tick/mite and lice prevalence than those in edge areas. *L. sahamalaza* tick/mite (estimate = −0.005, *df* = 0.030, *t* = −0.175, *P* = 0.974), lice (estimate = −0.000, *df* = 11.001, *t* = −0.008, *P* = 0.997) and flea (estimate = −0.007, *df* = 2.388, *t* = −0.110, *P* = 0.921) prevalence did also not vary significantly among sites, with the only pairwise difference of significance being that of higher tick/mite presence in Ankarafa edge in comparison to Anabohazo core (Supplementary File S3). Tick/mite (Estimate = 0.076, *df* = 2.381, *t* = 1.012, *P* = 0.403), lice (estimate = −0.006, *df* = 22.850, *t* = −0.091, *P* = 0.928) and flea (estimate = 0.018, *df* = 2.840, *t* = 0.181, *P* = 0.869) prevalence did not vary significantly among sites for *C. medius*, nor were there any pairwise differences (Supplementary File S3). Tick/mite (estimate = 0.005, *df* = 0.000, *t* = 0.133, *P* = 1.000), lice (estimate = 0.031, *df* = 0.003, *t* = 0.314, *P* = 0.992) and flea (estimate = 0.039, *df* = 3.205, *t* = 0.557, *P* = 0.614) prevalence in *M. sambiranensis* did not vary significantly between Anabohazo core and edge areas (Supplementary File S3). Multiple ectoparasite group infection varied significantly among all sites for *M. zaza* (estimate = −19.650, *z* = −4385.480, *P* < 0.001), with significantly higher numbers of individuals having multiple ectoparasite group infection in the edge areas of both Anabohazo and Ankarafa in comparison to core areas (Table 4, Supplementary File S4). However, there were no significant differences in multiple ectoparasite group infection between any sites for *L. sahamalaza* (estimate = −22.385, *z* = −0.025, *P* = 0.980) and *C. medius* (estimate = 0.000, *z* = 0.000, *P* = 1.000), nor between the core and edge areas of Anabohazo for *M. sambiranensis* (estimate = 0.000, *z* = 0.000, *P* = 1.000). Similarly to body condition, the effects of sex on ectoparasite prevalence appear to be significant in *M. zaza*, *C. medius* and *M. sambiranensis*, but not for *L. sahamalaza* (Supplementary File S3).

**Table 4:** The number of individuals sampled (including recaptures), the presence percentage of ticks/mites, lice and fleas and the presence percentage of multiple (>1) parasite groups in *Mirza zaza*, *Lepilemur sahamalaza*, *Cheirogaleus medius* and *Microcebus sambiranensis* in core and edge areas of Anabohazo and Ankarafa forest in the Sahamalaza-Iles Radama National Park of north west Madagascar

		Site			
Species	Measurement	Anabohazo Core	Anabohazo Edge	Ankarafa Core	Ankarafa Edge
*Mirza zaza*	*N*	81	29	53	25
	Tick/mite presence (%)	4.94^a^	24.13^a^	3.77^a^	24.00^a^
	Lice presence (%)	8.64^a^	34.48^a^	9.43^a^	44.00^a^
	Flea presence (%)	8.64^a^	17.24^a^	16.98^a^	28.00^a^
	Multiple group presence (%)	3.70^a^	17.24^b^	0.00^a^	28.00^b^
*Lepilemur sahamalaza*	*N*	12	8	16	15
	Tick/mite presence (%)	0.00^a^	0.00^a^	0.00^a^	33.00^a^
	Lice presence (%)	0.00^a^	25.00^a^	0.00^a^	13.33^a^
	Flea presence (%)	0.00^a^	25.00^a^	25.00^a^	13.33^a^
	Multiple group presence (%)	0.00^a^	12.50^a^	0.00^a^	6.66^a^
*Cheirogaleus medius*	*N*	4	5	9	9
	Tick/mite presence (%)	0.00^a^	0.00^a^	0.00^a^	44.44^a^
	Lice presence (%)	0.00^a^	0.00^a^	11.11^a^	44.44^a^
	Flea presence (%)	25.00^a^	0.00^a^	11.11^a^	22.22^a^
	Multiple group presence (%)	0.00^a^	0.00^a^	11.11^a^	22.22^a^
*Microcebus sambiranensis*	*N*	7	16	-	-
	Tick/mite presence (%)	0.00^a^	18.75^a^	-	-
	Lice presence (%)	12.50^a^	18.75^a^	-	-
	Flea presence (%)	12.50^a^	31.25^a^	-	-
	Multiple group presence (%)	0.00^a^	18.75^a^	-	-

Values with the same superscript letter within a row are not significantly different from each other in following GLMM analysis and pairwise comparisons. Data were collected between 6 March 2019 and 15 May 2022.

## Discussion

### Body condition

**Figure 2:**
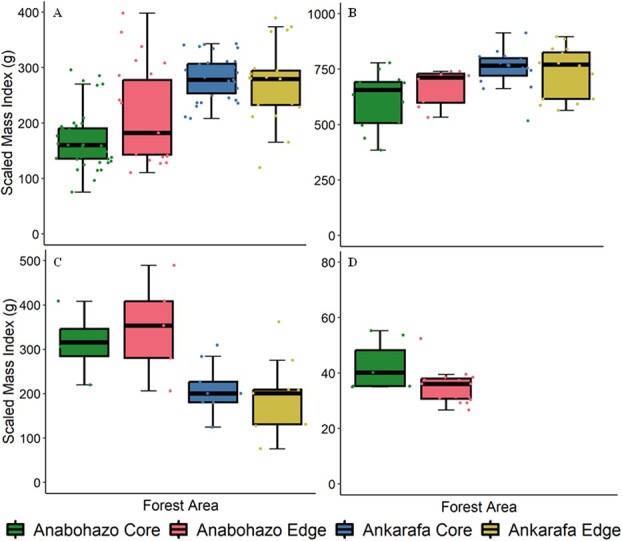
Summary of the scaled body mass index (g) of *Mirza zaza* (A), *Lepilemur sahamalaza* (B), *Cheirogaleus medius* (C) and *Microcebus sambiranensis* (D) in core and edge areas of Anabohazo and Ankarafa forest in the Sahamalaza-Iles Radama National Park of north west Madagascar. Boxplots display the median, interquartile ranges and minimum and maximum values excluding outliers. Dots represent individual lemurs. Data were collected between 6 March 2019 and 15 May 2022.

In contrast to our original prediction of less variability in Cheirogaleidae body condition in comparison to *L. sahamalaza*, results varied significantly among study areas for all four species. Further, our observations of increased body condition in Ankarafa forest for *L. sahamalaza* opposed our original prediction. Body condition of primates is frequently reported to deteriorate in fragmented and degraded habitats (Dunham *et al.*, 2008; Singleton *et al.*, 2015; Steffens and Lehman, 2016), but we observed the opposite for both *M. zaza* and *L. sahamalaza* in this study (Table 2, Fig. 2). This trend could be due to two different factors. Firstly, the abundance of preferential food items (*M. zaza*: fruit, flowers and invertebrates, *L. sahamalaza*: leaves of *Ficus* spp.*, Mangifera indica* and *Sorindeia madagascariensis*, Hending *et al.*, 2024) for these two species may be higher in fragmented and edge forest than in continuous and core habitat; plant and invertebrate communities do vary significantly between the surveyed areas (Hending *et al.*, 2024). For *L. sahamalaza*, this explanation is also supported by significantly higher densities of this species observed in Ankarafa in comparison to Anabohazo (Hending *et al.,* in press). Secondly, as both species require secure sleeping sites (*M. zaza*: dense, well-covered vegetation tangles, *L. sahamalaza*: secure tree holes) with specific characteristics for day resting (Seiler *et al.*, 2013; Rode-Margono *et al.*, 2016), and suitable sleeping sites may be more abundant in the secondary growth vegetation of Ankarafa in comparison to the larger trees of Anabohazo (Hending *et al.*, 2023a). *M. sambiranensis* body condition was observed to be higher in the core of Anabohazo in comparison to the edge (Table 2, Fig. 2). Mouse lemurs often thrive in edge and degraded habitat (Knoop *et al.*, 2018; Andriatsitohaina *et al.*, 2020), and these observations contrast with the literature (Burke and Lehman, 2015). However, body condition may be lower in these habitats due to food availability and increased competition resulting from higher densities of conspecifics (Zimmermann *et al.*, 1998; Hending *et al.*, in press).

As expected, *C. medius* body condition was higher in continuous forest in comparison to fragmented forest (Table 2, Fig. 2). This species must maintain good physiological health so that it can survive long periods of torpor (Fietz and Dausmann, 2006), and it relies on a diet of sugary fruits to achieve this (Fietz and Ganzhorn, 1999). These food items, along with secure tree holes necessary for torpor (Dausmann, 2012), are more-abundant in core forest in SIRNP in comparison to edge habitat (Hending *et al.*, 2024), which may be an explanatory factor for our observations here. Whilst we captured *C. medius* individuals during both the pre (January–April) and post (August–October) hibernation periods in both forests, and while we controlled for seasonal effects in the analysis, the impact of the hibernation period may be the greatest cause of body condition differences rather than fragmentation and edge effects. Season had an evident effect on body condition (Supplementary File S2), and whilst our results may suggest fragmentation and edge effect impacts on *C. medius*, these results must be interpreted cautiously and further work is required to clarify the body condition—fragmentation relationship in dwarf lemurs.

Sex had a significant effect on body condition in the three cheirogaleid species, but not for *L. sahamalaza* (Supplementary File S2). Sex has previously been documented to influence body condition in mouse and dwarf lemurs (Rakotoniaina *et al.,* 2016), and this observation is likely due to sex-specific feeding behavior and reproductive state throughout the study period which would strongly influence mass (Rasoazanabary, 2006; Radespiel *et al.*, 2022). Sex-specific behavioral ecology and its impacts on physiological are less profound in sportive lemurs (Mandl *et al.*, 2018; Bethge *et al.*, 2021), which may explain our result for *L. sahamalaza* here. It is worth noting here that lemurs were not sedated when body measurements were recorded; whilst all lemurs were easily restrained, this may have introduced some small imprecision to the measurement data.

### Fur condition scores

Until now, fragmentation effects on fur condition scores had not been studied in any nocturnal lemurs, but this metric has the potential to be an important indicator of health change within wild populations (Jolly, 2009a). Fur condition scores only differed among sites for *L. sahamalaza*, and scores were highest in fragmented edge habitat (Table 3). In contrast, tail fur condition scores were more variable and varied significantly among study areas in *M. zaza*, *L. sahamalaza* and *C. medius* (Table 3). Tail fur condition scores may have been more variable than fur as both inter and intra specific competitors and predators often grab arboreal and quadrupedal mammals by their tails during aggressive encounters, fighting, or attacking from behind (Fan and Jiang, 2009; Mincer and Russo, 2020). Predators of lemurs in SIRNP include hawks, owls, snakes and mammals (Fichtel, 2016). Higher variation in tail fur condition scores may therefore have been due to high fur scores in individuals that have either been involved in aggressive altercations with competitors or that have evaded predators after being captured, and such a measure could be an indicator of increased predation pressures. Tail scores were higher in fragmented and edge areas than in continuous core forest for *M. zaza*, *L. sahamalaza* and *C. medius* (Table 3); this may suggest that these populations are at higher risk of predation, something commonly reported for primates in fragments and edge habitats (Estrada and Coates-Estrada, 1996; Irwin *et al.*, 2009; Lenz *et al.*, 2014). It must be noted however that decreased fur condition as a result of predation and aggressive encounters, and also due to nutritional deficiencies, may not necessarily be a direct result of fragmentation, and so these results should be taken cautiously.

Whilst primate fur condition scores have not been linked directly to habitat-related factors (but see Crawford *et al.*, 2015), fur condition scores are known to be an indicator of disease and infirmity (Berg *et al.*, 2009), which may themselves be caused by habitat-related factors such as food availability (Chapman *et al.*, 2015) and parasite presence (Chapman *et al.*, 2006). Higher fur condition scores (i.e. poorer fur quality) could therefore be regarded as an indirect consequence of habitat degradation. If so, our results suggest that fragmentation, edge effects and habitat degradation has a negative impact on the health of *M. zaza*, *L. sahamalaza* and *C. medius*, all of which had higher body or tail fur scores in edge and fragmented areas in comparison to core and continuous areas.

### Ectoparasite prevalence

There were few differences in the prevalence of individual parasite groups between sites for all species. The only exceptions to this were higher tick/mite prevalence in edge habitat for *M. zaza* and in Ankarafa edge for *L. sahamalaza* (Table 4). While *M. zaza* also had higher multiple group infection rates in edge habitats, such rates did not differ between sites for the other three species (Table 4). As increased parasite prevalence in primates has been reported for fragmented and edge habitats previously (Gillespie *et al.,* 2005; Chapman *et al.,* 2006; Gillespie and Chapman, 2008; Mbora and McPeek, 2009; Schwitzer *et al.,* 2011), our significant pairwise results are as expected and conform to our original predictions. Fragmentation and edge effects increase the environmental stress of potential ectoparasite hosts and therefore their susceptibility to ectoparasite infestations (Kiene *et al.,* 2020; Kiene *et al.,* 2021), and the abundance of primate parasites may be elevated in edge habitat due to a range of environmental, climatic and anthropogenic factors (Chapman *et al.,* 2006; Marquès Gomila *et al.,* 2023). The higher abundance of parasites and the increased susceptibility to them may therefore explain our observations of higher tick/mite prevalence in edge habitat for *M. zaza* and *L. sahamalaza*. However, tick abundance and diversity, and indeed overall lemur parasite diversity, are sometimes lower in fragmented and edge habitats compared to core continuous forest (Kiene *et al.,* 2020), and isolated populations in threatened habitats often harbor fewer parasites (Altizer *et al.,* 2007). Further, ticks have an affinity for dark and humid microhabitats; lemur resting trees have these characteristics, and it is therefore in these tree holes that the ticks attach themselves to their lemur hosts (Hokan *et al.,* 2017; Bethge *et al.,* 2023). As parasite diversity and abundance (Kiene *et al.,* 2020) and the number of secure resting holes (Dausmann, 2012) are often higher in core continuous forest, other factors are the likely cause of higher tick prevalence in *M. zaza* and *L. sahamalaza* and the higher rates of multiple group infection in *M. zaza*. This may be due to increased cross-species transfer in edge habitats (Mbora and McPeek, 2009) and in fragments that have less occupiable space (crowding effect); in these habitats, the lemurs may come into direct contact with each other more frequently due to crowding (Gestich *et al.,* 2022).

Although difficult to control for in an observational study such as this, grooming also needs to be considered as a potential driver of ectoparasite prevalence, as parasites may spread more easily in lemur populations where all-grooming occurs (but see Duboscq *et al.,* 2016). However, the amount of time that these species engage in grooming and other social activity has not been found to vary significantly between fragmented and continuous forest or between edge and core areas (Hending *et al.,* 2024).

Similar to body condition, sex has previously been documented to influence parasite prevalence in mouse and dwarf lemurs (Rakotoniaina *et al.,* 2016; Rafalinirina *et al.,* 2019), and this observation is also likely the result of sex-specific behaviors and activities that may facilitate parasite transfer. It must be noted that our ectoparasite dataset was zero-heavy, and low sample sizes were recorded for *C. medius* and *M. sambiranensis*. Further sampling effort is therefore required to draw robust conclusions about the impacts of fragmentation and edge effects on ectoparasite prevalence in these species. The presence and abundance of ectoparasites at a site can also be strongly influenced by season (Rodriguez *et al.,* 2015), and further investigation into the seasonal impacts of ectoparasite prevalence in these lemurs is also required. While not always significant, season had a pronounced effect on the prevalence of some of the different ectoparasite taxon groups across species (Supplementary File S3). Our results must consequently be treated cautiously, and further work is required to expand on our study and determine whether fragmentation effects or season most strongly influence ectoparasite prevalence in these lemurs.

### Fragmentation effects on physiology

Interestingly, the impact of forest fragmentation and edge effects on the three investigated physiological variables differed. The physiological health of animals living in fragmented and degraded forests or edge and anthropogenic habitats is often reported as being negatively affected across all measured variables (Sauther *et al.,* 2006; Junge *et al.,* 2011; Singleton *et al.,* 2015) or unaffected overall (Junge and Louis, 2005; Burke and Lehman, 2015; Rakotoniaina *et al.,* 2016). However, when multiple variables have been investigated, single components of physiology have also been reported to be affected by degraded habitat in varying ways, while other components are simultaneously unaffected (Eley *et al.,* 1989). This appears to be the pattern observed in our study. Ecological factors that vary between fragmented and unfragmented forest and between core and edge habitats such as food availability (Asensio *et al.,* 2009; Mandl *et al.,* 2018), vegetation structure (Harper *et al.,* 2005; Hending *et al.,* 2023a) and microclimate (Didham and Lawton, 1999; Magnago *et al.,* 2015) have all been reported to impact single components of primate physiology such as body mass (Irwin *et al.,* 2007; Andriatsitohaina *et al.,* 2020), parasite prevalence (Raharivololona and Ganzhorn, 2009; Schwitzer *et al.,* 2011) and stress level (Rimbach *et al.,* 2013; Rakotoniaina *et al.,* 2016). It is likely that our results are due to specific responses of physiological health to specific environmental stressors (Killen *et al.,* 2013). We must reiterate here that our observations are preliminary, and further studies investigating multiple variables are required to determine whether these effects are conclusively the result of fragmentation and edge effects or are more associated with other factors (season, inter/intra-specific competition, etc.). Our work also needs to be expanded upon to determine whether fragmentation and habitat degradation effects on animal physiology are component-specific, whether fragmentation effects on different physiological variables are correlated and whether fragmentation and edge effects impact physiology synergistically.

### Future directions, conservation implications and conclusions

In conclusion, the body condition of the nocturnal lemur community within SIRNP appears to be significantly affected by forest fragmentation and edge effects, with two species responding positively to fragmentation and edge effects (*M. zaza* and *L. sahamalaza*) and two responding negatively (*C. medius* and *M. sambiranensis*). However, fur condition scores and ectoparasite prevalence appear to be affected to a lesser extent; significant negative responses to fragmentation and edge effects were observed in only *M. zaza* and *L. sahamalaza* for these two variables. Physiological responses to fragmentation and edge effects appear to be species-specific within lemurs, and responses also appear to be variable-specific. These findings are likely due to the specific ecology of each species and their flexibility to adapt to degraded habitats (Seiler *et al.,* 2013; Hending, 2021). Nocturnal lemurs are often reported to inhabit fragmented, degraded and anthropogenic habitats (Dinsmore *et al.,* 2016; Hending *et al.,* 2018; Knoop *et al.,* 2018; Schüßler *et al.,* 2018; Webber *et al.,* 2019). While our study suggests that these habitats may result in decreased physiological health, the behavioral plasticity and flexibility of these lemurs likely allow them to overcome such issues and thrive in these sub-optimal environments (Ganzhorn, 1993; Radespiel, 2006; Hending, 2021). Additional work is required to determine why certain aspects of lemur physiology are affected by environmental stressors while others remain unaffected. Further, although we controlled for seasonal effects in this study, nocturnal lemur physiology is known to be strongly correlated with seasonal factors such as food availability and forest phenology (Ganzhorn, 2002; Randrianambinina *et al.,* 2003; Raharivololona and Ganzhorn, 2010; Bethge *et al.,* 2023), and the relationship between season and physiology should be further investigated for lemurs in SIRNP.

Disregarding their flexibility in fragmented and degraded environments, all lemurs and indeed most primates require forest habitat to maintain viable populations (Cowlishaw and Dunbar, 2021). Over 80% of Madagascar’s original forest has already been lost, and the remaining patches continue to be threatened by deforestation, anthropogenic interference and unmitigated climate change (Harper *et al.,* 2007; Vieilledent *et al.,* 2018; Hending *et al.,* 2022b). Urgent conservation is now required to safeguard the future of these forests, as without them, lemurs face inevitable extinction (Schwitzer *et al.,* 2013).

## Supplementary Material

Web_Material_coae042

## Data Availability

The associated data for this manuscript can be obtained from the corresponding author upon reasonable request.
