# Supplementary material for: Forest fragmentation and edge effects impact body condition, fur condition and ectoparasite prevalence in a nocturnal lemur community
Source: Conserv Physiol. 2024 Jul 2;12(1):coae042. doi: 10.1093/conphys/coae042 (PMC11217907; doi:10.1093/conphys/coae042)
Supplement: Web_Material_coae042 [file web_material_coae042.zip › Supplementary_File_1_-_Forest_Fragment_Information (1).pdf]

Supplementary File 1: Location, total area, forest edge and forest core area and shape index (Patton, 1975) of Anabohazo Forest and 10 forest fragments of Ankarafa Forest chosen for inclusion in this study. Measurements were computed using GPS data obtained during forest perimeter walks (Cotton *et al*., Unpublished Data) in ArcGIS 10.6 (Esri, Redlands CA, USA).

**Forest**	**Fragment**	**Centre Latitude**	**Centre Longitude**	**Total Area (Ha)**	**Edge Buffer Area (Ha)**	**Forest Edge (%)**	**Area of Forest Core (Ha)**	**Forest Core (%)**	**Shape Index (SI)**
Anabohazo	-	-14.314	47.911	1168.81	290.54	24.86	878.27	75.14	1.577
Ankarafa	Ambodiamontana	-14.387	47.772	192.26	123.82	64.40	68.44	35.60	2.015
	Angodorabe	-14.373	47.742	247.22	132.91	53.76	114.31	46.24	2.075
	Ankarafa I	-14.373	47.763	69.70	62.97	90.34	6.73	9.66	2.283
	Ankarafa II	-14.381	47.758	18.31	18.31	100.00	0.00	0.00	1.598
	Bekotika	-14.385	47.757	65.26	55.47	85.00	9.79	15.00	1.530
	Bepamandry	-14.366	47.758	84.83	77.11	90.90	7.72	9.10	2.313
	Bidoroko	-14.371	47.778	120.45	104.14	86.46	16.31	13.54	2.770
	Lavakola	-14.392	47.762	50.62	46.64	92.14	3.98	7.86	1.664
	Meleintena	-14.377	47.754	61.93	56.95	91.96	4.98	8.04	1.945
	Tsangambato	-14.383	47.745	65.06	55.15	84.76	9.91	15.24	1.648
